# Habitual Coffee Consumption Enhances Attention and Vigilance in Hemodialysis Patients

**DOI:** 10.1155/2014/707460

**Published:** 2014-05-05

**Authors:** Petar M. Nikić, Branislav R. Andrić, Biljana B. Stojimirović, Jasna Trbojevic-Stanković, Zoran Bukumirić

**Affiliations:** ^1^Special Hospital for Cerebrovascular Disease “Sveti Sava”, Nemanjina 2, 11000 Belgrade, Serbia; ^2^Department of Nephrology, General Hospital, Health Center, 37000 Krusevac, Serbia; ^3^Medical Faculty, University of Belgrade, Institute of Nephrology, Clinical Centre of Serbia, 11000 Belgrade, Serbia; ^4^Department of Nephrology, Clinical Center “DR Dragisa Misovic”, 11000 Belgrade, Serbia; ^5^Medical Faculty, University of Belgrade, Institute of Statistics and Informatics, 11000 Belgrade, Serbia

## Abstract

*Objective.* Coffee drinking is the main source of caffeine intake among adult population in the western world. It has been reported that low to moderate caffeine intake has beneficial effect on alertness and cognitive functions in healthy subjects. The aim of this study is to evaluate the impact of habitual coffee consumption on cognitive function in hemodialysis patients. *Methods.* In a cross-sectional study, 86 patients from a single-dialysis centre underwent assessment by the Montreal Cognitive Assessment tool and evaluation for symptoms of fatigue, mood, and sleep disorders by well-validated questionnaires. The habitual coffee use and the average daily caffeine intake were estimated by participants' response to a dietary questionnaire. *Results.* Sixty-seven subjects (78%) consumed black coffee daily, mostly in low to moderate dose. Cognitive impairment was found in three-quarters of tested patients. Normal mental performance was more often in habitual coffee users (25% versus 16%). Regular coffee drinkers achieved higher mean scores on all tested cognitive domains, but a significant positive correlation was found only for items that measure attention and concentration (*P* = 0.024). *Conclusions.* Moderate caffeine intake by habitual coffee consumption could have beneficial impact on cognitive function in hemodialysis patients due to selective enhancement of attention and vigilance.

## 1. Introduction

Chronic kidney disease (CKD) is a growing health problem, and the prevalence of end-stage renal disease (ESRD) is steadily increasing worldwide. Coffee is the most widely consumed artificial beverage and is the main source of caffeine intake among adult population in the western world. Almost 80% of American adults drink coffee daily and nearly 75% of the total caffeine intake in US population comes from consumption of black coffee [1]. Coffee contains more than 1000 compounds, and habitual drinking has been associated with a number of harmful and beneficial effects on health. Caffeine (1,3,7-trimethylxanthine) is the most important and most extensively studied compound in coffee. The presumed stimulant effect of caffeine on physiological and mental states accounts for the habit-forming nature of coffee and is the main reason for the growing popularity of caffeinated beverages.

The amount of caffeine available in prepared beverage is variable and principally depends on a proportion of the mix between two primary types of coffee beans, the preparation method, and the size of the coffee cup. It is estimated that ingestion of a single cup of coffee provides doses from 0.4 to 2.5 mg of caffeine per kilogram. In humans caffeine absorption from stomach and intestine is rapid and almost complete, with peak blood levels occurring about 15–120 minutes after oral ingestion. Due to its hydrophobic properties, caffeine passes all biological membranes, including blood-brain barrier. The elimination occurs primarily by metabolism in the liver and less than 5% of caffeine is recovered unchanged in urine. Individual clearance rates of caffeine are variable and depend on various factors. For doses lower than 10 mg/kg, the half-life of caffeine ranges from two and a half hours to four and a half hours, with no significant difference between young and elderly humans [2]. Following normal human consumption, the only relevant pharmacological action of caffeine is mainly the antagonism of A1 and A2A adenosine receptors and, to a far lesser degree, the A2B and A3 receptors [3]. Adenosine as a general inhibitor of neuronal activity is closely involved in the regulation of arousal, sleep, and cognition. Therefore, the ability of caffeine to reduce adenosine transmission in the brain may be an effective mean to increase arousal level and cognitive performance in humans [4]. A recent meta-analysis of clinical studies on healthy subjects reported benefits of caffeine consumption on attention, psychomotor performance, mood, and cognitive functioning [5]. It is reported that regular caffeine consumption could prevent age-related cognitive impairment and may be inversely associated with the incidence of common neurodegenerative disease such as Alzheimer's and Parkinson's disease [6–8].

Due to aging population and raising frequency of risk factors for chronic renal failure (i.e., hypertension and diabetes mellitus) there is worldwide increase in the incidence of CKD, with an expected exponential growth in the number of patients with ESRD on renal replacement therapy in the near future. CKD is an independent risk factor for cognitive impairment with an inverse relationship between estimated glomerular filtration rate and prevalence of cognitive decline [9]. In patients on hemodialysis cognitive impairment is common with more than one-third showing signs of severe cognitive deficit, almost three times more than the age-matched general population [10, 11]. Cognitive impairment and dementia are associated with an increased mortality, decreased quality of life, more frequent hospitalizations, and inappropriate decision making in dialysis patients [12]. Given the widespread popularity of coffee drinking and the growing number of uremic patients with cognitive impairment, even potentially small benefits of caffeine intake may have important implications for the health and quality of life in this vulnerable group.

The aim of this study is to explore the effect of habitual coffee consumption and regular caffeine intake on cognitive function in patients on maintenance hemodialysis. Because the cognitive performance in uremic subjects could be influenced by various dialysis-associated disorders, we have also examined the effect of regular caffeine intake by coffee drinking on the number of conditions that may have impact on cognitive function.

## 2. Methods

### 2.1. Study Participants

The initial sample consisted of all 101 patients with ESRD, receiving conventional hemodialysis therapy for minimum three months, from the community hospital dialysis facility in central Serbia. Study candidates were at least 18 years of age and underwent standard intermittent hemodialysis using bicarbonate solution and capillary dialyzers three times per week for 4 hours each day. Major exclusion criteria include (a) unstable medical condition or confusion precluding the testing on more than two occasions; (b) inadequate visual and hearing acuity or other relevant sensorimotor impairment; (c) acute or chronic psychosis; and (d) presence of significant predialysis cognitive impairment. Fifteen patients were excluded from the study on the basis of the exclusion criteria. Thus, the remaining 86 patients were recruited for this study. The study was approved by local research ethic committee, and informed consent was obtained from all patients.

### 2.2. Demographic Factors, Comorbidity, and Medication Data

Demographic data were obtained from the medical record and by self-report. Laboratory analyses were obtained within a month before testing. Education was recorded as completed years of schooling and further subdivided into four levels. Regarding smoking habits, subjects were classified as current smoker (one or more cigarettes per day), and nonsmoker. History of stroke was based on the clinical symptoms, neurological examination, and computed tomography or magnetic resonance imaging of the brain. Hypertension was defined as blood pressure ≥140/90 mm Hg or history of antihypertensive medication. In addition, use of the following medications was recorded for each subject: benzodiazepines, antidepressants, anticonvulsants, opioid analgesics, combined simple analgesics with caffeine, and H1-receptor antagonists. No patients had used the methylxanthine drugs like theophylline or doxofylline on regular basis.

### 2.3. Assessment of Cognitive Function

In all subjects the cognitive examination was performed in the first hour of dialysis, about 48 hours from the start of a previous hemodialysis session. In the patients who felt unwell or were unable to complete testing on the scheduled date, the examination was repeated during the next available dialysis session. In individuals with sight impairment some tests were administered orally.

For the screening of cognitive function we used the Montreal Cognitive Assessment test (MoCA), due to its high sensitivity to conditions associated with mild cognitive impairment and to executive dysfunction caused by frontal subcortical damage. Its sensitivity is 90% for detecting mild cognitive impairment and 100% for detecting dementia, compared with 18% and 87% for the minimental state examination [13]. The cut-off sore of 26 (one point is added for subjects with less than 12 years of education) is suggested for detection of mild cognitive impairment [14, 15]. Test scoring of visuoconstructional/executive functions was corrected for simple drawing errors in seven subjects using the nondominant hand. Definitions of the specific MoCA domains are as follows. Visuospatial abilities are assessed using a clock-drawing task and a three-dimensional cube copy (max 4 points). Executive functions are assessed using the Trail making task part B, a phonemic fluency task, and a two-item verbal abstraction task (max 4 points). Attention, concentration, and working memory are evaluated using a sustained attention task, a serial subtraction task, and digits forward and backwards (max 6 points). Language domain is assessed using a three-item confrontation naming task with low-familiarity animals, repetition of two syntactically complex sentences, and the fluency task (max 6 points). The short-term memory recall task involves two learning trials of five nouns and delayed recall after approximately 5 minutes (max 5 points). Final task is the orientation of study participants to time and place (max 6 points) [15].

### 2.4. Assessment of Depression, Anxiety, Fatigue, and Sleep Disturbances

In order to evaluate the presence and possible influence of various mental states on cognitive performance, each study participant was asked to complete a battery of self-reported tests measuring symptoms of depression, anxiety, fatigue, and sleep disturbances. The Beck Depression Inventory-II (BDI), a well-validated 21-item self-administered questionnaire, is one of the most widely used measures of depressive symptoms. Higher scores (range 0–63) indicate more severe depressive effect. It was reported that scores ≥16 maximize sensitivity and specificity for depressive symptoms in patients with ESRD [16, 17]. To determine a participant's anxiety level we used Beck Anxiety Inventory (BAI), a 21-item self-report multiple-choice questionnaire, which measures the severity of anxiety on a scale from 0 to 3. A total score of 8 to 15 points is interpreted as a mild level of anxiety, and subjects scoring 16 or more points show moderate to severe anxiety symptoms with typical somatic side effects. For assessing the intensity of fatigue we used the FACIT Fatigue Scale, one of health related, quality of life questionnaires devised for the management of chronic illness referred to as the FACIT Measurement System. The FACIT tool is a short 13-item instrument that measures on a four-point Likert scale (4 = not at all fatigued to 0 = very fatigued) the level of fatigue during a subject's usual daily activities over the past week. The total score ranges from 0 to 52 points, and the overall result is negatively associated with the level of fatigue (i.e., high score indicates less fatigue) [18]. The general level of recent subjective sleepiness in our patients with ESRD was assessed by the Epworth Sleepiness Scale (ESS), an 8-item self-report measure of sleepiness on Likert 4-point scale, with a range of possible scores from 0 to 24. A score of 11 or more points is reported to indicate significant level of subjective sleepiness [19]. The possible presence of clinically significant insomnia was assessed by the Athens Insomnia Scale (AIS). The AIS is an 8-item instrument which measures the most prevalent symptoms of insomnia and potentially negative effect of disturbed night sleep on daytime activities during the last month. Soldatos suggested a cut-off score of 10 as predictive for significant insomnia symptoms [20]. For the evaluation of subjective sleep quality, we used the Pittsburgh Sleep Quality Index (PSQI), the tool designed to assess overall sleep quality in clinical samples. The PSQI consists of 19 self-rated questions, mainly as 4-point Likert scale, and five items rated by the bed partner about habitual sleep during a 1-month interval. A global PSQI score has a possible range of 0 to 21 points, with higher scores indicating worse sleep quality. A total score greater than 5 indicates poor quality of sleep [21].

### 2.5. Assessment of Coffee Drinking and Caffeine Intake

We estimated participants' average daily caffeine intake by their response to a dietary questionnaire about the habitual consumption of coffee and other caffeinated products during the previous year. Since none of the responders reported the regular intake of other caffeinated beverages or food besides black coffee, we equated its using with the total amount of caffeine. Those study subjects drinking at least one cup of coffee per day were further asked to classify their usual method of preparing coffee and the size of serving cup. We assessed the total intake of caffeine by summing the caffeine content for a specific amount, depending on the preparation method and cup size, multiplied by the frequency of its use. The estimated caffeine content per single cup was 120 mg, based on data about type and an average serving size of brewed black coffee consumed in Serbia.

### 2.6. Statistical Analysis

Statistical analysis was carried out using the SPSS 18 software (SPSS, Chicago, IL, USA). Summarizing data are displayed as mean and standard deviation (SD) if not otherwise specified. The Kolmogorov-Smirnov test was used to assess normal distribution of data. Continuous normally distributed variables were compared using Student's *t*-test or the Mann-Whitney *U* test for nonnormally distributed variables. Differences among categorical variables were analyzed using the chi-square test or the two-tailed Fisher's exact test, as appropriate. Baseline characteristics of eligible hemodialysis patients who participated in the study and the patients who met the exclusion criteria were compared using chi-square tests and *t*-tests as appropriate. Cognitive impairment on the MoCA test was determined using raw scores and published cutoff scores, and afterwards the separate cognitive domains were used in comparing the group findings. Due to sample size limitations, the main variables obtained on tests measuring sleep disturbances (AIS, ESS, and PSQI), a severity of fatigue (FACIT), and psychiatry symptoms (BDI and BAI) were first analyzed using total scores and subsequently dichotomized based on published cut-off scores. *P*  values less than .05 were considered statistically significant.

## 3. Results

More than three-quarters (78%) of the eighty-six eligible study participants consumed black coffee on a regular basis. In the group of sixty-seven regular coffee drinkers, low use (1 cup/day) was reported by 36% (*n* = 24), moderate use (2 cups/day) by 40% (*n* = 27), and intake of more than 2 cups a day by 24% (*n* = 16) of the examined patients. Only two patients (3%) have reported heavy coffee use (≥5 cups/day) on a regular basis. The estimated adjusted amount of consumed caffeine, which almost exclusively came through ingestion of separate portions of black coffee, was 3.4 mg/kg/day (250 mg). There was no statistical significant difference in the habitual coffee drinking and total amount of ingested caffeine comparing fifteen patients who met exclusion criteria and the patients included in the study. More than 90% of habitual users in our study reported drinking coffee predominantly in the morning and early afternoon, which is followed by overnight abstinence. There were no significant differences in demographics, clinical characteristics, lifestyle factors, and selected medical comorbidities between persons who drink coffee daily and noncoffee users. Prevalence of subjects with less than 8 years of education did not differ substantially between noncoffee and regular coffee drinkers. Group comparisons are shown in Table 1.

A quarter of tested subjects demonstrate normal cognitive performance as assessed by MoCA tool (*n* = 21; 24.4%). A higher percentage of patients with a cognitive score within normal limits were observed in the group of habitual coffee users, but the prevalence of cognitive impairment using established cut-off value did not significantly differ between the two groups. The patients who did not regularly use coffee tended to have more severe symptoms of daily sleepiness and insomnia, and these associations reach statistical significance for clinical insomnia and daytime sleepiness on AIS and ESS scales, respectively. However, the overall quality of sleep measured by PSQI inventory was not significantly different between the two groups (*P* = 0.728). There was no significant impact of the regular coffee intake compared to the noncoffee use on ratings of mental fatigue (*P* = 0.745). Concerning the effects of habitual dietary caffeine intake on severity of anxiety, the subjects in our study reported a mild level of anxiety symptoms. The noncaffeine users had the higher self-reported baseline anxiety level, but the mean BAI score was not statistically different between groups (12.6 ± 11.4 versus 11.2 ± 8.7; *P* = 0.606). Clinically significant depressive symptoms, identified by adjusted cut-off score for diagnosis of depression in ESRD, were more often present in patients who did not drink coffee (*P* = 0.045) (Table 2).

Influence of regular caffeine consumption on combined MoCA task items is shown in Table 3. Habitual coffee users had higher mean scores on all tested cognitive domains, but significant positive correlation was found only for items that measure attention, concentration, and working memory (*P* = 0.024).

## 4. Discussion

We did not find substantial difference in demographic and clinical variables regarding the habitual coffee consumption in patients with ESRD. The result from our study, where more than three-quarters of the tested subjects shown impaired cognitive performance, confirmed the high frequency of cognitive deficit (30–87%) found in dialysis population [10, 22–24]. Insomnia and daytime sleepiness were common sleep complaints, and almost 80% of patients in our study reported poor global sleep quality. However, the overall quality of sleep was not significantly different between the nonregular and habitual coffee users, and this is in accordance with other studies that explore relationship between caffeine intakes and sleep quality [25, 26]. Although there is evidence that caffeine by augmentation of dopamine function in striato-thalamo-cortical system may counteract the effect of low arousal found in fatigued subjects, we did not find significant impact of the regular coffee intake on ratings of mental fatigue in our study participants [4, 27]. This is probably because the effects of caffeine on central fatigue are highly dependent on underlying arousal levels and situations in which additional demands were placed on visual information processing [28]. The intensity of depressive affect and prevalence of clinical relevant depressive symptoms were significantly lower in our study participants who were habitual coffee consumers. A recent meta-analysis reported a significant negative association between depression severity and cognitive performance, particularly with regard to episodic memory, executive function, and processing speed [29].

The mean total score on MoCA testing was on average two points higher in subjects with regular caffeine intake, and habitual coffee users had higher mean scores on all tested cognitive domains. However, significant positive correlation was found only for tasks that measure attention, concentration, and working memory. The majority of tested subjects showed severe impairment across multiple aspects of executive functions. Despite the fact that our finding shows a beneficial, albeit small, impact of the regular caffeine consumption on executive functions, there was no significant difference between groups regarding the coffee consumption routine. Our data indicates substantial positive effect of moderate habitual coffee drinking and regular caffeine intake on the attention system. This is clinically relevant because the patients with ESRD had significant deficit on measures of attention [30]. Attention and concentration help us to select and focus on what is important by modifying neural activity in specific cortical areas, thus augmenting the cerebral response to particular perceptual stimuli [31]. Caffeine influences stimulus processing and enhances vigilance by increasing cortical activation and the rate at which information accumulates. Thus, the regular caffeine intake by coffee drinking may effectively modulate the encoding and long term retrieval of memories by enhancing sustained attention [32, 33]. A recent study performed on 160 healthy volunteers confirmed an enhancing effect of caffeine on memory consolidation [34]. Furthermore, the beneficial effect of caffeine under states of reduced alertness is proved to be quite consistent and the ability of caffeine to increases or sustain attention may be important for patients undergoing dialysis treatment [35, 36]. Considering the gender difference, the early short-term activation by caffeine in a low dose (100 mg) was observed to be greater in young healthy males [37]. However, due to small number of females in our group of noncaffeine users, we were not able to further explore the influence of gender on cognition and mood.

An issue that was not addressed in this study was whether there is dose response relationship between caffeine intake and cognitive function. The latter was due to a fact that the majority of our regular coffee users consume low to moderate dose of caffeine. Nevertheless, despite the likely wide variation in the caffeine content and the timing of coffee consumption, the majority of studies did not support caffeine dose-response relationship. Furthermore, it was found that doses as low as 37.5 mg, even in first exposure to caffeine, enhance cognitive function [5]. The possible bias in responses due to the hemodialysis procedure on the hepatic metabolism of caffeine could not be excluded, but the shortness of average half-life of caffeine limits the implication of its serum level on cognitive and mental functions.

Several limitations to this pilot study need to be acknowledged. First, the number of patients, especially in the group of nonregular coffee users, was relatively small, and it was not possible to further investigate the confounding effect of the various mental conditions (i.e., depression, mental fatigue, and sleep disorders) on the results of cognitive testing. Second, the results of testing may be influenced by the dialysis procedure itself or by the time of examination. Also, the predictive value of cognitive screening test is dependent on the prior probability of cognitive impairment in the population studied, and can be manipulated by changing the cut-off value. Third, we tried to include as many conditions as possible that may have an impact on cognitive and mental performances in the dialysis population, but there are many other unrecognized medical disorders that may acutely or chronically in negative manner affect mental status. Fourth, because the study was cross-sectional we could not determine the cause-effect relationship between the regular caffeine consumption and cognitive performance. In future studies it would be prudent to compare the caffeine-related implications of regular coffee consumption on key aspects of cognitive functions on groups of chronic patients with a similar degree of cognitive impairment as the patients with chronic renal failure (e.g., patients with small vessel brain disease, dementia, etc.), along with healthy control subjects, as well. The strengths of this study include the well-defined single center subject sample, a comprehensive estimation of cognitive function, and standardized evaluations of mood state, sleep disturbance, and level of fatigue. In addition, all MoCA evaluations were administered by a single physician.

Finally, it should be noted that, even though in public perception and our paper the terms coffee and caffeine are interchangeable, the biologic effects of coffee cannot be reduced to the isolated effects of caffeine that it contains [38].

## 5. Conclusions

Hemodialysis patients frequently have cognitive impairment with marked executive dysfunction and reduced attention due to defective processing of relevant perceptual stimulus. The findings from this pilot study suggest that the observed beneficial impact of moderate caffeine intake by habitual coffee consumption on cognitive performance is principally due to direct enhancement of attention, concentration, and vigilance. Further research regarding the role of coffee and its main agent caffeine in patients on renal replacement therapy would be of great importance since their neuroprotective, stimulatory, and mental activating effects may be especially important in this vulnerable group.

## Figures and Tables

**Table 1:** Demographic and clinical characteristics of the noncoffee users and habitual coffee users on maintenance hemodialysis.

	Noncoffee users (*n* = 19)	Habitual coffee users (*n* = 67)	*P*
Age (years)	63.0 ± 10.1	58.8 ± 11.5	0.156
Sex, *n* (%)			
Male Female	16 (84.2%) 3 (15.8%)	44 (65.7%) 23 (34.3%)	0.120
Education level (years), *n* (%)			
None (0–4) Primary (8) Secondary (12) High (≥14)	4 (21.1%) 7 (36.8%) 5 (26.3%) 3 (15.8%)	12 (17.9%) 21 (31.3%) 26 (38.8%) 8 (11.9%)	0.694
Duration of HD (mo), median (range)	47.0 (8.0–282.0)	59.0 (4.0–334.0)	0.135
Shift, *n* (%)			
Morning Afternoon	10 (52.6%) 9 (47.4%)	32 (47.8%) 35 (52.2%)	0.708
Current smoker, *n* (%)	4 (21.1%)	12 (17.9%)	0.746
Psychotropic medication use, *n* (%)	8 (42.1)	28 (41.8)	0.980
Previous stroke, *n* (%)	1 (5.3%)	6 (9.0%)	1.000
Hypertension present, *n* (%)	17 (89.5%)	56 (83.6%)	0.724
Hematocrit, (%)	32.5 ± 3.4	33.4 ± 4.4	0.451
iPTH, (ng/L), median (range)	129.5 (8.3–566.0)	143.5 (6.0–2500.0)	0.699

iPTH: intact parathyroid hormone; HD: hemodialysis; data presented as mean ± SD if not otherwise specified; to convert from the SI unit to the conventional unit, divide by the following conversion factor: intact parathyroid hormone = 1.0 (pg/mL).

**Table 2:** The cognitive function, neuropsychiatric symptoms, severity of fatigue, and sleep disturbance relative to habitual coffee intake in ESRD patients.

	Noncoffee users	Habitual coffee users	*P*
Montreal cognitive assessment, total points	20.3 ± 6.3	22.3 ± 4.3	0.190
MoCA score ≥26, *n* (%)	3 (15.8%)	18 (26.9%)	0.382
FACIT fatigue score	29.2 ± 11.6	30.1 ± 10.2	0.745
Athens Insomnia Scale, total points	10.7 ± 5.9	6.7 ± 5.5	0.010
Athens Insomnia Scale, score >6, *n* (%)	15 (83.3%)	30 (50.8%)	0.014
ESS total score, median (range)	9.0 (0.0–21.0)	5.0 (0.0–24.0)	0.019
ESS score ≥10, *n* (%)	8 (44.4%)	12 (20.7%)	0.048
PSQI, total points	9.7 ± 5.6	7.3 ± 3.6	0.128
PSQI, score ≤5, *n* (%)	4 (26.7%)	12 (20.7%)	0.728
Beck Depression Inventory II, total points	19.7 ± 15.3	11.0 ± 05.6	0.048
Beck Depression Inventory II, score ≥16, *n* (%)	8 (53.3%)	15 (25.9%)	0.045
The Beck Anxiety Inventory, total points	12.6 ± 11.4	11.2 ± 8.7	0.606

FACIT: The functional assessment of chronic illness therapy fatigue scale; ESS: the epworth sleepiness scale; PSQI: pittsburgh sleep quality index.

Data presented as mean ± SD If not otherwise specified.

**Table 3:** Influence of habitual caffeine consumption on cognitive performance in multiple domains on MoCA.

MoCA cognitive domain	Noncoffee users (*n* = 19)	Habitual coffee users (*n* = 67)	*P*
MoCA, total score median	22.0 (7–28)	23.0 (11–29)	0.190
Visuospatial abilities	2.3 ± 1.6 (0–4)	2.7 ± 1.4 (0–4)	0.225
Executive functions	1.3 ± 1.2 (0–4)	1.8 ± 1.2 (0–4)	0.102
Attention	4.2 ± 1.5 (1–6)	4.9 ± 1.1 (0–6)	0.024
Language	4.6 ± 1.1 (2–6)	4.9 ± 1.1 (1–6)	0.333
The short-term memory recall task	1.9 ± 1.5 (0–5)	2.0 ± 1.5 (0–5)	0.805
Orientation	5.5 ± 1.0 (3–6)	5.8 ± 0.5 (4–6)	0.193

Values are expressed as mean ± SD (range).
